# Focal Traumatic Brain Injury Impairs the Integrity of the Basement Membrane of Hindlimb Muscle Fibers Revealed by Extracellular Matrix Immunoreactivity

**DOI:** 10.3390/life14050543

**Published:** 2024-04-24

**Authors:** Mette Albæk Kristensen, Karen Kalhøj Rich, Tobias Christian Mogensen, Andreas Malmquist Damsgaard Jensen, Åsa Fex Svenningsen, Mengliang Zhang

**Affiliations:** 1 Department of Molecular Medicine, University of Southern Denmark, DK-5230 Odense, Denmark; mettealbaekkristensen@hotmail.com (M.A.K.); krich@health.sdu.dk (K.K.R.); tcmogensen@health.sdu.dk (T.C.M.); aasvenningsen@health.sdu.dk (Å.F.S.); 2 Brain Research—Inter Disciplinary Guided Excellence (BRIDGE), University of Southern Denmark, DK-5230 Odense, Denmark

**Keywords:** traumatic brain injury, hindlimb postural asymmetry, motor deficits, extracellular matrix, laminin, collagen type IV

## Abstract

Traumatic brain injury (TBI) stands as a prominent global cause of disability, with motor deficits being a common consequence. Despite its widespread impact, the precise pathological mechanisms underlying motor deficits after TBI remain elusive**.** In this study, hindlimb postural asymmetry (HL-PA) development in rats subjected to focal TBI was investigated to explore the potential roles of collagen IV and laminin within the extracellular matrix (ECM) of selected hindlimb muscles in the emergence of motor deficits following TBI. A focal TBI was induced by ablating the left sensorimotor cortex in rats and motor deficits were assessed by measuring HL-PA. The expression of laminin and collagen IV in eight selected muscles on each side of the hindlimbs from both TBI- and sham-operated rats were studied using immunohistochemistry and semi-quantitatively analyzed. The results indicated that the TBI rats exhibited HL-PA, characterized by flexion of the contralateral (right) hindlimb. In the sham-operated rats, the immunoreactive components of laminin and collagen IV were evenly and smoothly distributed along the border of the muscle fibers in all the investigated muscles. In contrast, in the TBI rats, the pattern was broken into aggregated, granule-like, immunoreactive components. Such a labeling pattern was detected in all the investigated muscles both from the contra- and ipsilateral sides of the TBI rats. However, in TBI rats, most of the muscles from the contralateral hindlimb showed a significantly increased expression of these two proteins in comparison with those from the ipsilateral hindlimb. In comparison to sham-operated rats, there was a significant increase in laminin and collagen IV expression in various contralateral hindlimb muscles in the TBI rats. These findings suggest potential implications of laminin and collagen IV in the development of motor deficits following a focal TBI.

## 1. Introduction

Traumatic brain injury (TBI) is a significant contributor to both mortality and disability, affecting an estimated 64–74 million individuals worldwide annually [1]. TBI is associated with persistent impairments, including emotional instability, memory, and attention deficits, as well as sensorimotor issues [2]. Animal studies have demonstrated that injuries to the sensorimotor cortex result in motor function deficits in the contralateral musculature [3,4]. Moreover, conditions affecting the upper motor neurons, such as TBI, stroke, and cerebral palsy (CP), lead to sensorimotor impairments, like spasticity and contractures [5,6,7,8,9]. Spasticity is defined as a velocity-dependent increase in muscle resistance to passive stretch, and muscle contractures, causing increased passive muscle stiffness and restricted joint motion without active force, are common motor deficits following TBI [10,11]. The precise pathological mechanisms contributing to motor deficits are not yet fully understood. It has been suggested that the heightened passive muscle stiffness after upper motor neuron lesions results from modifications of the elastic elements within the muscle fibers and/or in the extracellular matrix (ECM) [12].

Previous investigations into potential mechanisms contributing to contracture development in children with CP have suggested the involvement of ECM composition changes in increased passive stiffness in CP muscle contractures [11,13,14]. Specifically, these studies have shown a correlation between increased total collagen content and CP severity [14]. Furthermore, the deposition of laminin, another ECM component, is also increased in CP muscle [11].

Laminin and collagen are the major proteins found in the ECM of skeletal muscles [15,16,17]. The basement membrane, the ECM layer, which is connected to the sarcolemma, primarily consists of collagen IV, while laminin-211 is the most prevalent non-collagenous protein [18,19,20,21]. Both laminin-211 and collagen IV play crucial roles in maintaining skeletal muscle homeostasis and functions. It has previously been demonstrated that mutations in the laminin-ɑ2 chain-encoding gene lead to partial or complete laminin-ɑ2 chain deficiency, causing congenital muscular dystrophy (CMD) [22,23]. Additionally, mutations in the collagen IV-encoding gene, *Col4a1*, have been associated with muscular dystrophy, characterized by muscle fiber atrophy, fibrosis, and ECM remodeling [24].

The role of ECM in the emergence of motor deficits after TBI has not previously been explored. CP and TBI share similar motor symptoms resulting from upper motor neuron damage. We therefore hypothesized that changes in the ECM, as revealed in studies investigating the pathological mechanisms leading to contracture development in children with CP [11,13,14], could influence motor deficits following TBI.

In this study, we investigated the induction of hindlimb postural asymmetry (HL-PA) in a well-established unilateral TBI rat model, where the left sensorimotor cortex was ablated to assess the development of motor deficits [3,4]. We also examined whether the immunoreactivity of laminin and collagen IV in the ECM of eight different muscles in both the contra- and ipsilateral hindlimbs would be impacted, with the purpose of evaluating their potential contribution to the development of motor deficits following TBI.

## 2. Materials and Methods

Adult male Sprague Dawley rats (Janvier, Denmark) weighing 382–513 g were used in this study. The animals were kept in a 12 h day–night cycle at a constant environmental temperature of 21 °C (humidity: 65%) and received water and food ad libitum. The animals were randomly assigned to their respective experimental groups (TBI or sham surgery). The animal experiments followed the guidelines of the European Union (EU) Directive 2010/63/EU. Approval of the animal experiments was obtained from the Animal Experiments Inspectorate (Authority No.: 2019-15-0201-0015).

### 2.1. Traumatic Brain Injury and Sham Animal Model

Prior to the experiments, 14 rats were randomly divided into one of the two groups: TBI or sham group (n = 7/group). However, 4 rats died due to unexpected reasons either before (1 rat) or after (3 rats) the surgery. Thus, only 5 rats in each group were utilized for the present study. One hour prior to surgery, an oral administration of 0.2 mg/kg buprenorphine (Temgesic, CAS: 52485-79-7; Indivior Europe, Dublin, Ireland) was given to the rats. The analgesic was applied as a post-operative pain reliever and acted over 24 h. The rats were anesthetized subcutaneously with a solution of 50 mg/mL ketamine (Ketaminol Vet., CAS: 6740-88-1; MSD Animal Health, Stockholm, Sweden) and 20 mg/mL xylazine (Rompun Vet., CAS: 7361-61-7; Elanco Denmark, Ballerup, Denmark). The surgical area of the skull was shaved and disinfected before surgery. Lidocaine (Xylocain, CAS: 137-58-6; Aspen Nordic, Ballerup, Denmark) was administered subcutaneously on the surgical area and in the ears as a local analgesic. To prevent dryness of the eyes, 2 mg/g carbomer (Viscotears, CAS: 9007-20-9; Bausch and Lomb Nordic AB, Stockholm, Sweden) was applied on the eyes.

**Figure 1:**
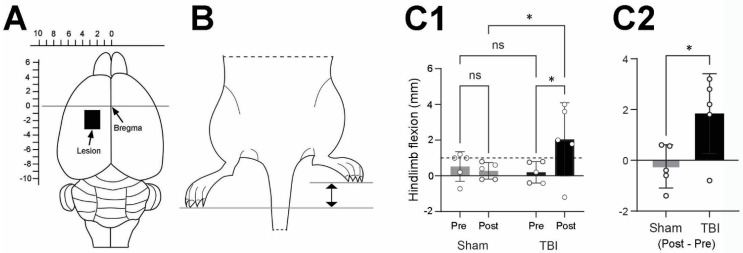
Lesion site and hindlimb postural asymmetry (HL-PA) analysis of the traumatic brain injury (TBI)- and sham-operated rats. (**A**) Schematic drawing showing the intended TBI site on the left cortex (black rectangle). Actual lesion sites may have varied slightly from rat to rat. Scales on top and left of the brain are the distance relative to the bregma (mm). (**B**) Schematic drawing showing that the left TBI caused flexion of the right hindlimb. The difference between the projections of the longest digits towards the midline from the right and left paws was measured as the magnitude of HL-PA (between the two arrows). (**C1**) Bar graph showing the mean (±SD) magnitude of hindlimb flexion (the difference between right and left hindlimb flexion) from all the sham and TBI rats pre- and post-operation (Pre and Post). A positive number indicates flexion of the right hindlimb and a negative number indicates flexion of the left hindlimb. The dash line indicates the 1 mm threshold for HL-PA. Two-way ANOVA followed by uncorrected Fisher’s LSD post hoc analyses indicated there was a statistically significant difference between pre- and post-operation in TBI rats and between sham and TBI rats post-operation. (**C2**) Bar graph showing the changes in magnitude of hindlimb flexion post-operation in relation to pre-operation in both sham and TBI groups (Post–Pre). Unpaired *t*-test shows a significant difference between sham and TBI groups. * *p* ≤ 0.05; ns: not significant.

The TBI and sham operations were performed as previously described [3,4]. In short, the TBI operation was executed on a stereotaxic holder under a surgical microscope. Following exposure of the skull, bregma was identified and its coordinates notated. The sensorimotor area was identified and marked with the coordinates 1.8–3.8 mm laterally of the midline on the left parietal bone and 0.5–4.0 mm posterior to the bregma (Figure 1A). The marked area of the skull was removed by drilling. The dura was opened, and the gray matter of the left sensorimotor cortex was ablated with a glass pipette connected to an electrical suction machine (Craft Duo-Vec Suction Unit; Rocket Medical Plc, Watford, UK). Following completion of the ablation, the bleeding was stopped using Spongoston (Ethicon, Raritan, Raritan, NJ, USA). The wound was sutured with a 3-0 Vicryl suture (Article No.: V393H; Ethicon, Raritan, NJ, USA) and lidocaine (Xylocain, CAS: 137-58-6; Aspen Nordic, Ballerup, Denmark) was applied directly on the surgical area. The same anesthetic and operative procedure were applied for the sham operation; however, the dura remained intact, and the sensorimotor cortex was not removed. Following the surgery, the rats were housed in a heat cabinet for 24 h at a constant temperature of 26 °C and henceforth housed at a constant temperature of 21 °C until the end of the experiment.

### 2.2. Analysis of Hindlimb Postural Asymmetry (HL-PA)

The HL-PA was measured prior to surgery and 14 days after TBI or sham operation. The assessment of HL-PA has previously been described [3,4]. Briefly, the rats were subcutaneously anesthetized using a ketamine xylazine solution (0.25 mL/100 g bodyweight). The measurements were initiated once the rats no longer exhibited any reflexes. The animals were placed in a prone and symmetric position on a millimeter grid paper sheet, and both hindlimbs were equally pulled 5–10 mm and then released. HL-PA was measured as the millimetric difference observed between the longest digits of the left and right hindlimb paws (Figure 1B). The procedure was performed five times in immediate succession for each rat, and the averaged value was used to generate a HL-PA for the individual rat.

### 2.3. Tissue Collection and Muscle Tissue Preparation

Under the anesthesia following HL-PA measurement, the rats were euthanized by decapitation. The brains were visually examined to make sure there was no brain injury in the sham-operated rats and the injury was in the correct place for the TBI rats. After that, the brains were snap-frozen and stored for further use. The following hindlimb muscles from the left and right hindlimbs of the TBI- and sham-operated rats were collected 14 days after surgery: extensor digitorum longus (EDL), peroneus longus (PL), triceps surae which included the lateral head of gastrocnemius (LG), medial head of gastrocnemius (MG), and soleus (SOL), biceps femoris (BF), semitendinosus (ST), and vastus lateralis (VL). These muscles were a selection of flexors, extensors, and bifunctional muscles and covered muscle from both the leg and the thigh (Table 1).

The muscles from both the TBI- and sham-operated rats were collected 14 days after operation following measurements of the postural asymmetry (Table 1). The muscles were extracted while the rats were under anesthesia with a ketamine xylazine solution (0.25 mL/100 g bodyweight). To ensure complete anesthetization during removal of the muscles, the animals received 0.62 mg/g sodium pentobarbital (Exagon Vet., CAS: 76-74-4; Salfarm, Kolding, Denmark) subcutaneously. The muscle tissue was cut into appropriate pieces and immediately placed into a tissue cassette and plunged into liquid nitrogen (AGA, CAS: 7727-37-9; Linde Gas, Odense, Denmark) for approximately 10 s (until the ceasing of bubbling). The tissue was stored at −80 °C until sectioning.

The fresh frozen muscle tissue was cut transversely into 30 µm sections with a Cryostat (CM3050S; Triolab, Leica, Brøndby, Denmark). The muscle tissue was placed on Superfrost plus adhesion microscope slides (VWR, Cat. No.: 631-0108; Søborg, Denmark) and stored at −80 °C.

### 2.4. Immunohistochemistry for Detection of Collagen IV and Laminin

The indirect immunohistochemistry (IHC) detection system, the avidin–biotin complex (ABC) method, was used to label laminin and collagen IV and thereby assess whether a TBI would alter the expression of these proteins in the ECM of selected hindlimb muscles.

The fresh frozen muscle sections were initially fixated in 4% paraformaldehyde (PFA) (CAS: 30525-89-4; Sigma-Aldrich, Søborg, Denmark) diluted in 0.01 M phosphate-buffered saline (PBS) for 10 min. The sections were incubated in 0.01 M PBS containing 0.3% H_2_O_2_ (CAS: 7722-84-1; Sigma-Aldrich, Søborg, Denmark) for 30 min to prevent endogenous peroxidase activity. The muscle tissue was blocked for unspecific binding by incubating the sections in blocking serum consisting of 5% goat serum (Cat No.: 16210-064; Gibco, Thermo Fisher Scientific, Odense, Denmark, or Cat. No.: SOOLW10501; Biowest, VWR, Søborg, Denmark) and 2% bovine serum albumin (CAS: 9048-46-8; Sigma-Aldrich, Søborg, Denmark) diluted in 0.01 M PBS for 1 h. The primary antibodies, 1:500 rabbit anti-laminin (REF: AB11575; Abcam, Cambridge, UK) and 1:500 rabbit anti-collagen IV (REF: AB6586; Abcam, Cambridge, UK) were diluted in the blocking serum and added to the sections for incubation for 48 h at 4 °C. Hereafter, the secondary antibody, 1:5000 goat anti-rabbit IgG (REF: SAB4600007; Sigma, Søborg, Denmark) was diluted in blocking serum and applied to the sections for 1 h. The tissue was afterwards incubated in ABC (REF: PK-6100; Vector Laboratories, Biozol, Germany) diluted in 0.01 M PBS. Laminin and collagen IV in the ECM were visualized by incubating the sections in 0.05 M Tris-buffered saline (TBS, pH = 7.2) with 0.05% 3,3′-diaminobenzidine tetrahydrochloride (CAS: 868272-85–9; Sigma-Aldrich, Søborg, Denmark) and 0.01% H_2_O_2_ for 8 min. The sections were finally rinsed in Milli-Q water and dehydrated in 70%, 96%, and 100% ethanol and subsequently in xylene (CAS: 1330-20-7; VWR, Søborg, Denmark). Lastly, the sections were coverslipped with DePeX mounting medium (CAS: 14208-10-7; VWR, Søborg, Denmark).

### 2.5. Data Acquisition and Analysis

The transverse muscle sections were imaged with a LEICA DM6000 microscope (connected to LEICA CTR6000) and pictures were obtained using a Leica DFC420 digital microscope camera and Leica Application Suite software (version 2.8.1) (all the hardware and software were from Leica Microsystems, Wetzlar, Germany). The images were taken with 10× magnification and at an 8-bit grayscale setting. The parameter settings of the software (exposure time, saturation, contrast, and gain) were maintained at the same values for all sections from the sham and TBI rats. The images used in the figures were further processed with Adobe Photoshop (Version 24.7.2; Adobe Inc., San Jose, CA, USA) to achieve a higher quality of the pictures.

The ImageJ software (Version 1.53k) was employed to achieve the area fraction (%) of laminin and collagen IV of a region of interest (usually a whole picture). Initially, all images were given a threshold to ensure that only pixels above the threshold were measured as laminin- or collagen IV-positive labeled elements. Pixels above the threshold were measured with the ImageJ software and provided as an area fraction of the total image. Statistical analyses were performed with the GraphPad Prism software (Version 10.2.1). Two-way ANOVA followed by uncorrected Fisher’s LSD post hoc analyses were performed to analyze the HL-PA score of the TBI- and sham-operated rats prior to surgery and 14 days after surgery (pre- and post-surgery) and to analyze the difference in the labeled area fraction between the left and right hindlimb muscles for the same operation group along with the difference between the TBI- and sham-operated rats for the same hindlimb. In addition, unpaired *t*-test was also used to analyze the changes in hindlimb flexion between the sham and TBI groups before and 14 days after surgery. The average area fraction of an individual group is expressed as the mean ± standard deviation (SD). The significance level is designated as *p* ≤ 0.05 for all statistical analyses.

One investigator was primarily responsible for animal allocation, surgery, HL-PA assessment, and ECM immunoreactive density analyses. ECM immunoreactive density analyses in selected muscles were also performed by a second investigator who was not aware of the animal grouping (blind). Since the results achieved from the two investigators showed the same trend, only the results from the first investigator are reported in this paper. Statistical analyses were performed twice with two different investigators.

## 3. Results

### 3.1. Development of Hindlimb Postural Asymmetry (HL-PA) following Traumatic Brain Injury

Figure 1C1 shows the HL-PA analysis of the TBI- and sham-operated rats before surgery (Pre) and 14 days after the operation (Post). The HL-PA analysis was based on the millimeter difference in the projection to the midline from the corresponding digits between the right and left hindlimbs. A hindlimb was regarded as flexed if it had a shorter projection than the other hindlimb. Furthermore, flexion of the ipsilateral hindlimb was given a negative score, whereas flexion of the contralateral hindlimb was given a positive score. A threshold value of 1 mm [25] was utilized to delineate the asymmetry of the hindlimbs. A HL-PA value below the threshold was thus regarded as symmetric while a value above 1 mm was recognized as asymmetric.

As shown in Figure 1C1, the sham-operated rats had a mean HL-PA value below the threshold value for asymmetry prior to and post-surgery, indicating symmetry of the ipsi- and contralateral hindlimbs. The sham-operated rats were therefore utilized as a control group to the TBI-operated rats in this study. The TBI-operated rats demonstrated a larger flexion of the contralateral hindlimb 14 days after surgery compared to before the operation (10.2-fold). The magnitude of HL-PA (2.04 mm) of the TBI-operated rats 14 days after surgery exceeded the applied threshold, indicating the development of contralateral hindlimb flexion of the brain-injured rats. Two-way ANOVA analysis followed by uncorrected Fisher’s post hoc testing showed that there was a statistically significant difference before and after surgery in the TBI rats (*p* = 0.0243) and between the TBI and sham rats post-surgery (*p* = 0.0301). Figure 1C2 indicated that the difference in the flexion changes pre- and post-operation between sham and TBI rats was statistically significant (*p* = 0.0315). The results indicated that the rats exposed to a focal TBI of the left sensorimotor cortex developed flexion of the contralateral hindlimb 14 days after surgery, while the sham-operated rats demonstrated hindlimb postural symmetry.

### 3.2. Traumatic Brain Injury Interrupts the Integrity of Laminin and Collagen IV Expression in the Basement Membrane of Hindlimb Muscle Fibers

**Figure 2:**
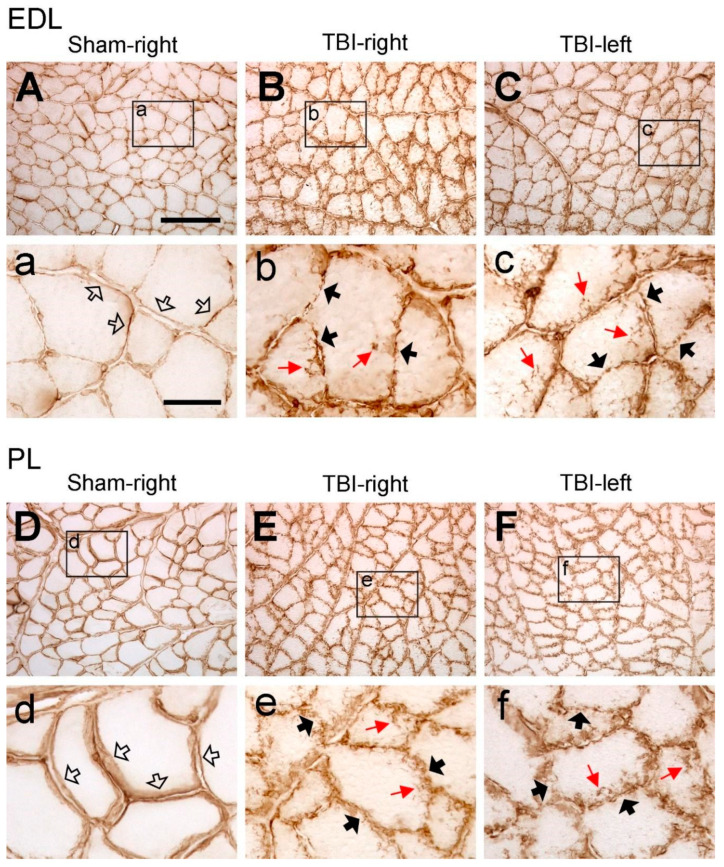
Example images of laminin immunoreactivity of extensor digitorum longus (EDL) and peroneus longus (PL) after sham surgery (Sham) or traumatic brain injury (TBI). For the sham-operated rats, only the pictures from the right (contralateral) side are shown (**A**,**D**) since the immunoreactivity from both sides was similar. For the TBI rats, pictures from both sides are shown (**C**–**F**). (**a**–**f**) are the enlargements of the area demarcated by the rectangle in (**A**–**F**), respectively. In the sham-operated rats, laminin-immunoreactive components were smoothly and evenly distributed along the sarcolemma (hollow arrows in **a**,**d**). However, in the TBI rats, the immunoreactive components were aggregated along the sarcolemma of muscle fibers and formed a granule-like labeling pattern (black arrows in **b**,**c**,**e**,**f**). The immunoreactive products were also seen to extend to the cytoplasm of the muscle fibers (red arrows in **b**,**c**,**e**,**f**). Scale bar in (**A**), valid for (**A**–**F**), 200 µm; in (**a**), valid for (**a**–**f**), 50 µm.

We first examined the expression of laminin and collagen IV systematically in all the collected muscles from the sham- and TBI-operated rats to see whether there were differences in the expression patterns in these animals. As exemplified in Figure 2A,D, in the muscles from the sham-operated rats, laminin immunoreactivity was located around the sarcolemma of the muscle fibers. The immunoreactive components were smoothly and evenly distributed around each muscle fiber, although the abundance of the immunoreactive components varied between different muscles. This labeling pattern was similar to the results that were reported in other studies in rodent hindlimb muscles [26,27], indicating that the sham surgery did not affect the expression pattern of laminin. However, upon examination of the muscles from the TBI rats, it was found that the pattern was disrupted, i.e., the smooth distribution pattern of the immunoreactive components was broken; instead, the immunoreactive components were aggregated along the sarcolemma of the muscle fibers and formed a granule-like labeling pattern (Figure 2B,C,E,F). The immunoreactive components were often seen to extend from the sarcolemma to the sarcoplasm of the muscle fibers, reflecting the internalization of laminin. Surprisingly, such a phenomenon was seen in the muscles both from the contralateral and ipsilateral hindlimbs of the brain-injured rats regardless of the muscle’s functionality (extensor or flexor). This result indicates that TBI not only causes pathological changes in the contralateral hindlimb muscles but also in the ipsilateral hindlimb muscles.

**Figure 3:**
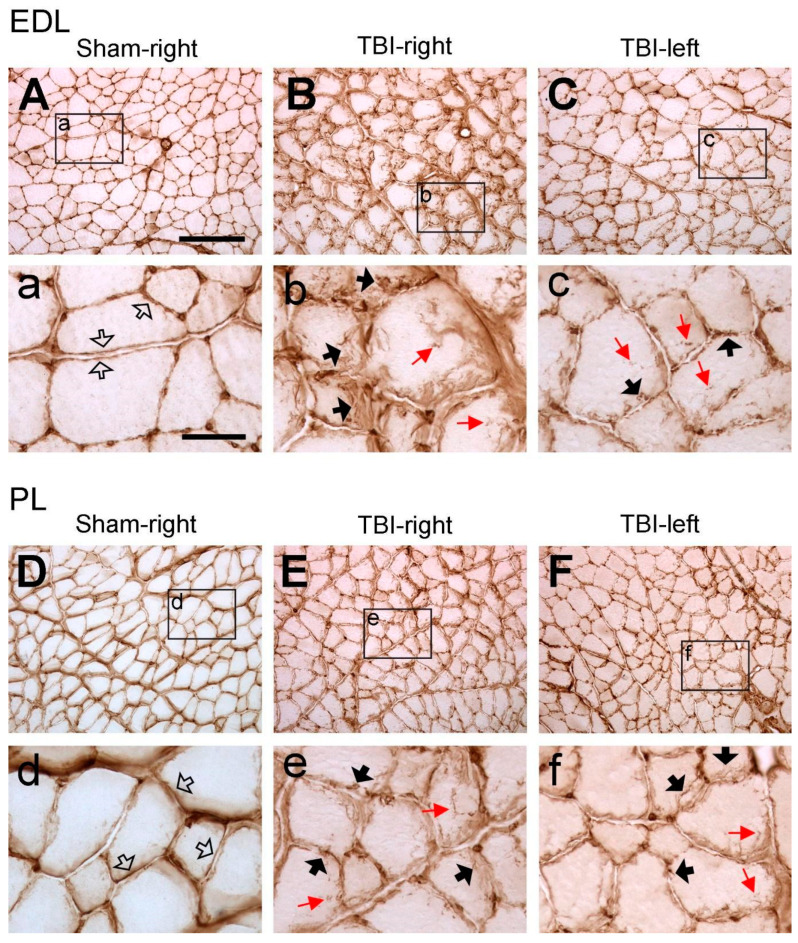
Example images of collagen IV immunoreactivity of extensor digitorum longus (EDL) and peroneus longus (PL) after sham surgery (Sham) or traumatic brain injury (TBI). For the sham-operated rats, only the images from the right (contralateral) side are shown (**A**,**D**) since the immunoreactivity from both sides was similar. For the TBI rats, images from both sides are shown (**C**–**F**). (**a**–**f**) are the enlargements of the area demarcated by the rectangle in (**A**–**F**), respectively. In the sham-operated rats, collagen IV-immunoreactive components were smoothly and evenly distributed along the sarcolemma (hollow arrows in **a**,**d**). However, in the TBI rats, the immunoreactive components were aggregated along the sarcolemma of muscle fibers and formed a fiber- or granule-like labeling pattern (black arrows in **b**,**c**,**e**,**f**). The immunoreactive products were also seen to extend to the cytoplasm of the muscle fibers (red arrows in **b**,**c**,**e**,**f**). Scale bar in (**A**), valid for (**A**–**F**), 200 µm; in (**a**), valid for (**a**–**f**), 50 µm.

Similar to the laminin immunolabeling on the sham-operated rats, collagen IV immunoreactivity was located around the sarcolemma of muscle fibers, and the immunoreactive components were smoothly and evenly distributed around each muscle fiber (Figure 3A,D). This labeling pattern is similar to the results that were reported in other studies in rodent hindlimb muscles [28,29], indicating that the sham surgery did not affect the expression pattern of collagen IV. In the muscles from the TBI rats, such a pattern was interrupted, forming a fiber- or granule-like labeling pattern and internalization of immunoreactive components, similar to the laminin immunolabeling. Such a phenomenon was also seen in the hindlimb muscles both from the contralateral and ipsilateral sides of both the extensor and flexor muscles, indicating detrimental effects of TBI on the muscles from both the contralateral and ipsilateral sides (Figure 3B,C,E,F).

### 3.3. Significant Increase in Laminin and Collagen IV Expression in the ECM of Multiple Contralateral Hindlimb Muscles following TBI

**Figure 4:**
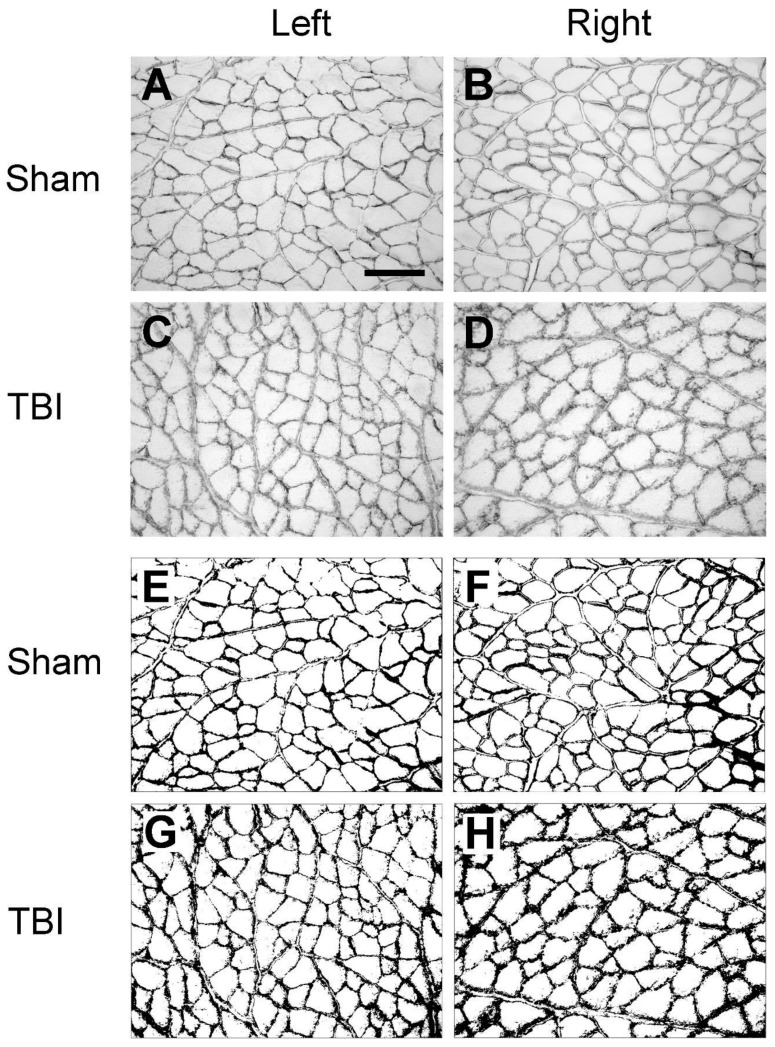
Example images of laminin immunoreactivity of semitendinosus (ST) after traumatic brain injury (TBI) or sham surgery. The images of the left and right hindlimb are from the same TBI or sham rat. (**A**–**D**) are the laminin immunoreactivity prior to threshold application. (**E**–**H**) are the same images after thresholding. From the threshold images, it appears that laminin-immunopositive components along the basement membrane of the muscle fibers are thicker and rougher in the contralateral (right) hindlimb (**H**) than in the ipsilateral (left) hindlimb (**G**) in the TBI rat and in either hindlimb of the sham rat (**E**,**F**), although in the ipsilateral hindlimb (**G**), the labeling is not as smooth as in the sham rat. Scale bar in (**A**), valid for all panels, 200 µm.

**Figure 5:**
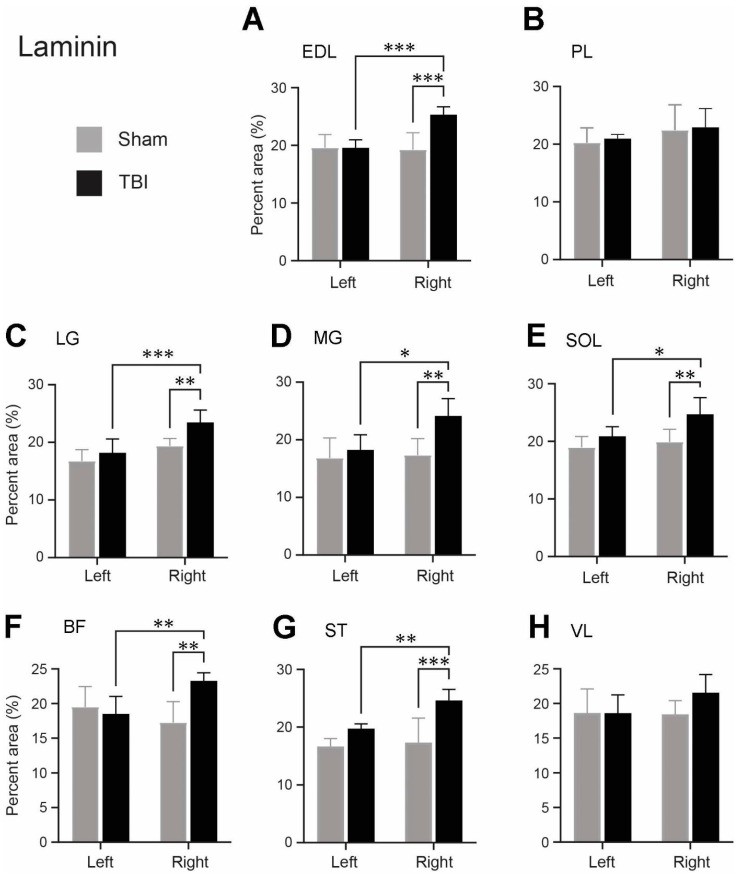
Area fractions (%) of the laminin-labeled areas of the hindlimb muscles of the traumatic brain injury (TBI)- and sham-operated rats (mean + SD). (**A**) extensor digitorum longus (EDL), (**B**) peroneus longus (PL), (**C**) lateral head of gastrocnemius (LG), (**D**) medial head of gastrocnemius (MG), (**E**) soleus (SOL), (**F**) biceps femoris (BF), (**G**) semitendinosus (ST), (**H**) vastus lateralis (VL) from the right and left hindlimbs of the TBI- and sham-operated rats. N = 5/group except for MG of the contralateral hindlimb (right) of the TBI-operated rats, where N = 4. * *p* ≤ 0.05, ** *p* ≤ 0.01, *** *p* ≤ 0.001, two-way ANOVA followed by uncorrected Fisher’s LSD post hoc analyses. The pairs with a non-significant difference were not labeled.

Since we observed that the density of the laminin and collagen IV immunoreactivity was different for the same muscles between the sham-operated and TBI rats, and between the ipsi- and contralateral hindlimbs of the TBI rats, we conducted an analysis of the immunoreactive densities of laminin and collagen IV. As illustrated in Figure 4, the laminin-immunoreactive density was analyzed on grayscale images in eight muscles on each side of the hindlimbs. The background immunoreaction was removed by applying a threshold to the grayscale images (Figure 4E–H). The area fraction of the laminin-labeled area of the right and left hindlimb muscles of the TBI- and sham-operated rats are presented in Figure 5A–H and are based on measurements from the threshold-applied images.

There was no significant difference in the laminin immunoreactivity between any of the hindlimb muscles of the sham-operated rats (Figure 5). The laminin expression in six muscles was, however, significantly increased in the right hindlimb compared to the same muscles of the left hindlimb of the TBI-operated rats: EDL (*p* < 0.001), LG (*p* < 0.001), MG (*p* = 0.0106), SOL (*p* = 0.0143), BF (*p* = 0.0085), and ST (*p* = 0.0065) (Figure 5A,C–G). Depending on the muscles, the laminin expression was 1.25–1.32 times elevated in the right hindlimb as opposed to the left hindlimb of the TBI-operated rats. The laminin immunoreactivity was also significantly increased in these muscles of the right hindlimb of the TBI-operated rats compared to the same muscles of the right hindlimb of the sham-operated rats with the *p* values being <0.001 (EDL and ST), 0.0052 (LG), 0.0042 (MG), 0.0034 (SOL), 0016 (BF), respectively (Figure 5A,C–G). The laminin expression was 1.21–1.42 times higher in these right hindlimb muscles of the TBI-operated rats compared to the right hindlimb of the sham rats. The muscles, PL and VL, did not show any significant difference between the contralateral hindlimb and ipsilateral hindlimb of the TBI-operated rats or between the right hindlimb of the TBI- and sham-operated rats (Figure 5B,H).

**Figure 6:**
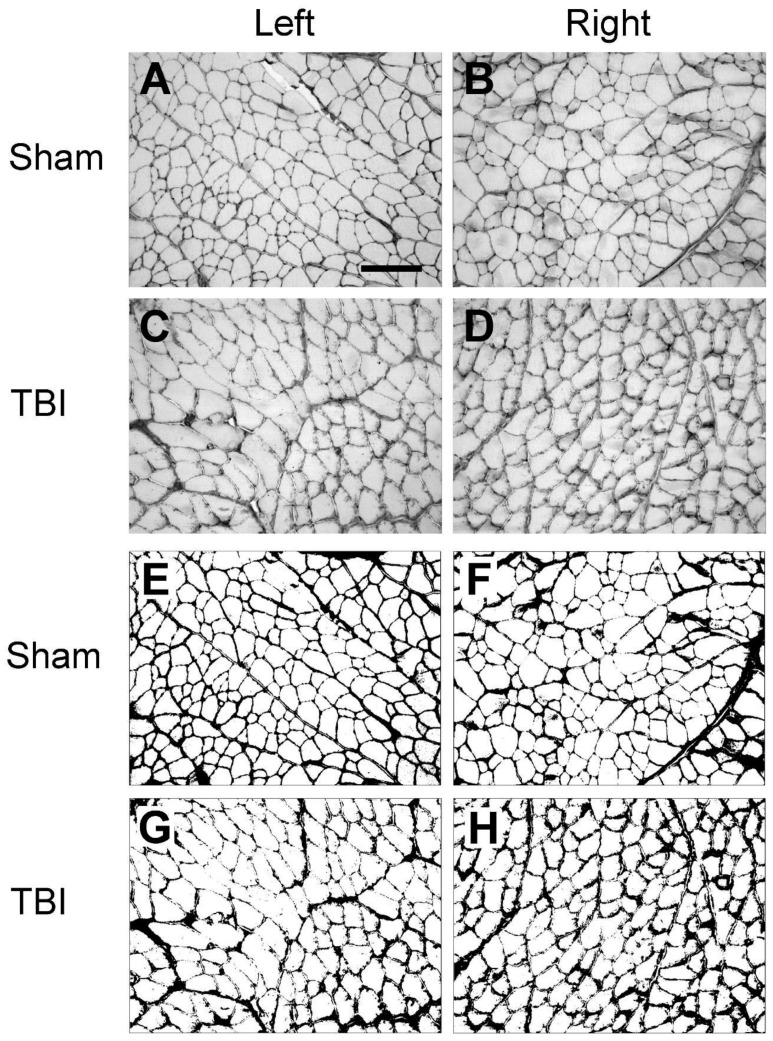
Example images of collagen IV immunoreactivity of extensor digitorum longus (EDL) after traumatic brain injury (TBI) or sham surgery (Sham). The images of the left and right hindlimbs are from the same TBI or sham rat. (**A**–**D**) are the collagen IV immunoreactivity prior to threshold application. (**E**–**H**) are the same images after thresholding. From the threshold images, it appears that collagen IV-immunoreactive components along the basement membrane of the muscle fibers are thicker and rougher in the contralateral (**right**) hindlimb (**H**) than in the ipsilateral (**left**) hindlimb (**G**) in the TBI rat and in either hindlimb of the sham rat (**E**,**F**), although in the ipsilateral hindlimb (**G**), the labeling is not as smooth as in the sham rat. Scale bar in (**A**), valid for all panels, 200 µm.

**Figure 7:**
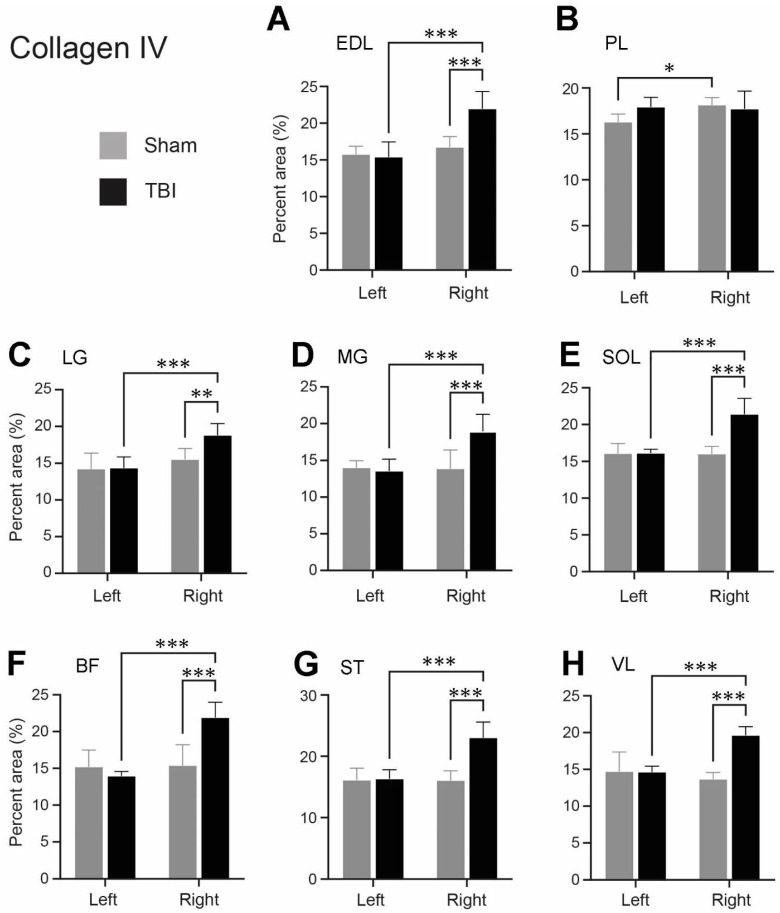
Area fractions (%) of the collagen IV-labeled areas of the hindlimb muscles of the traumatic brain injury (TBI)- and sham-operated rats (mean + SD). (**A**) extensor digitorum longus (EDL), (**B**) peroneus longus (PL), (**C**) lateral head of gastrocnemius (LG), (**D**) medial head of gastrocnemius (MG), (**E**) soleus (SOL), (**F**) biceps femoris (BF), (**G**) semitendinosus (ST), (**H**) vastus lateralis (VL) from the right and left hindlimbs of the TBI- and sham-operated rats. N = 5/group. * *p* ≤ 0.05, ** *p* ≤ 0.01, *** *p* ≤ 0.001, two-way ANOVA followed by uncorrected Fisher’s LSD post hoc analyses. The pairs with a non-significant difference were not labeled.

As for laminin, we also observed a significantly increased expression of collagen IV in the ECM of several contralateral hindlimb muscles following TBI. Figure 6 demonstrates the collagen IV immunoreactivity on grayscale images both before (Figure 6A–D) and after (Figure 6E–H) thresholding to eliminate the background immunoreaction. The measured area fractions of collagen IV of the eight hindlimb muscles from the TBI-and sham-operated rats after threshold application are presented in Figure 7.

Following sham surgery, the expression level of collagen IV between the corresponding muscles of the ipsi- and contralateral hindlimbs was unchanged, except for the PL, where the difference between the ipsilateral and contralateral sides was significant (*p* = 0.0316) (Figure 7). Following TBI surgery a statistically significant increase in the collagen IV expression was seen in the contralateral EDL, LG, MG, SOL, BF, ST, and VL muscles compared to the same muscles on the ipsilateral hindlimb, with *p* values ≤ 0.001 (Figure 7A,C–H). The density of collagen IV immunoreactivity in these seven muscles was 1.3- to 1.57-fold higher in the contralateral (right) hindlimb compared to the ipsilateral (left) hindlimb of the TBI-operated rats, depending on the muscle selected. For the same muscles, the collagen IV immunoreactivity was significantly increased in the contralateral (right) hindlimb of the TBI-operated rats compared to the contralateral (right) hindlimb of the sham-operated rats, as demonstrated in Figure 7A,C–H, with all *p* values being ≤ 0.001 except the LG, where *p* = 0.0074. Furthermore, depending on the specific muscle, the collagen IV mean area fraction was 1.21- to 1.44-fold higher in the contralateral (right) hindlimb of the TBI-operated rats as opposed to the contralateral (right) hindlimb of the sham-operated rats.

Finally, we conducted a correlation analysis between the amplitudes of HL-PA (post-operative values minus pre-operative values) and the fold changes in the expression of laminin and collagen IV in TBI rats (right side/left side). The results indicated no correlation between these two parameters (*p* > 0.05 for all the muscles of both ECM components) (Supplementary Figure S1).

## 4. Discussion

In this study, we demonstrated altered expression patterns of the two ECM proteins—laminin and collagen IV—in the examined hindlimb muscles bilaterally. We also observed a significantly increased expression of these proteins in most of the muscles on the contralateral hindlimb to the brain injury. The modifications in the ECM composition may have influenced the functionality of the associated muscles, potentially contributing to motor deficits in the hindlimbs and suggesting a development of hindlimb postural anomality following a TBI**.**

Hindlimb postural asymmetry (HL-PA), exceeding the predefined threshold of 1 mm, was observed in the contralateral hindlimb following ablation to the left sensorimotor cortex. The emergence of HL-PA in TBI rats is consistent with findings from our previous investigations using a right-side TBI rat model albeit with a slightly lower magnitude at the same time points [3]. The lower magnitude could be ascribed to methodological constraints, including variations in anesthesia and inconsistencies in the hindlimb traction procedure, or to biological variables including secondary brain injury effects rather than to disparities in the location of injury [30,31].

A new finding of the present study is the observed alteration in the expression patterns of the laminin and collagen IV across all investigated hindlimb muscles affecting both the ipsilateral and contralateral sides of the TBI rats. Especially, the immunoreactive components of these proteins transitioned from a smooth outline along the muscle fiber borders, as observed in the sham-operated rats, to a granule-like appearance in the TBI rats. This transformation suggests that a unilateral brain injury precipitates morphological changes in muscle tissue on both hindlimbs of the body. It is plausible that similar changes in the ECM expression could also manifest in muscles located in other parts of the body, such as the forelimbs. Remodeling of muscle ECM is indeed recognized as a common phenomenon as a reaction to many different factors, including exercise, disuse, aging, inflammation, and muscle damage [32,33]. In our study, the hindlimb muscles were not subjected to direct assault; consequently, the ECM remodeling was likely mediated via neural and/or humoral pathways. Pingel et al. utilized transmission electron microscopy to demonstrate that the basement membrane of the gastrocnemius muscle of children with CP is morphologically thicker and less defined compared to that of typically developing children [13]. The same study also identified several downregulated pathways in CP children, including laminin interactions and collagen fibril crosslinking. Taken together with our study, it is plausible to hypothesize that ECM remodeling might be involved in disruption of the muscle fiber integrity following damage to the upper motor neurons. Moreover, it is conceivable that additional proteins such as dystrophin and integrin may be dysregulated and thereby may have contributed to the observed alterations of the basement membrane morphology in our study.

It is conceivable that TBI induces plastic alterations in the spinal circuitry which in turn impacts the associated muscle activity [4,34]. Our previous study suggested that signaling from the brain injury may be transmitted via humoral pathways, as evidenced by motor deficits in a unilateral TBI model that was previously subjected to a complete spinal cord transection [25]. Determining whether these effects are attributable to one or both pathways needs to be further investigated.

Another interesting issue is what plastic changes or remodeling occur for different ECM components in the basement membrane following TBI. Several factors are involved in the maintenance of the normal muscle morphology and function, including laminin, collagen I, III, and IV, fibronectin, proteoglycan, nidogen, etc. [17]. Although only two components in the basement membrane were examined in this paper, it is likely that some of the other components may also undergo similar changes. We do not know what caused the granule-like shape of the laminin- and collagen IV-immunoreactive components. One possibility is that the originally evenly distributed fibers may have clumped together, or it could have resulted from structural alterations in other components. One such component, merosin (a laminin α-2 subunit), is a key ECM protein that forms a mechanical connection between the sarcolemma and collagen [35]. Should the structure of merosin be interrupted, it will likely lead to alterations in the configuration of collagen and other ECM components. Since we used a pan-laminin antibody, which should have also detected merosin, the observed disruption in the laminin-immunoreactive pattern may have also partially accounted for changes in merosin’s immunoreactivity. Further research is required to understand how the relationships between various ECM components are altered in response to brain injury, particularly utilizing super-resolution microscopy and electron microscopy.

In addition to morphological alterations, we found a significant increase in laminin expression in the ECM of BF, EDL, LG, MG, and ST muscles of the contralateral hindlimb compared with the ipsilateral hindlimb following a focal TBI. A similar increase in laminin expression was observed when comparing the contralateral hindlimb muscles (BF, EDL, LG, MG, ST, and SOL) from the TBI-operated rats with the corresponding side (right) of the hindlimb muscles from the sham-operated rats. Interestingly, similar results concerning increased laminin expression have previously been reported in the basement membrane in hamstring muscles of CP children [11]. Taken together, laminin appears to be implicated in muscle dysfunction related to damage to the upper motor neurons. It has also been suggested that upon muscle injury, the ECM undergoes remodeling with a significant and moderate upregulation of several laminin isoform-encoding genes in mice [36]. As muscle injury has shown to increase muscle stiffness [37], it could be speculated that laminin may be involved in the progress of muscle stiffness and perhaps motor deficits. Our study utilized a pan-laminin antibody; thus, the results are related to the total laminin expression at the basement membrane. The specific isoforms of laminin, e.g., laminin-211, expression levels that are altered following TBI should, thus, be further investigated.

Our findings also revealed a significant increase in collagen IV expression in the contralateral hindlimb muscles, including BF, EDL, LG, MG, SOL, ST, and VL, after ablating the left sensorimotor cortex in comparison with the muscles on the ipsilateral side of the TBI rats and the same side of the sham-operated rats. Similar results have been observed in studies examining the ECM composition in contracture muscles of children with CP. In them, the total collagen content of the ST and VL muscles was significantly higher in the CP patients compared to typically developing children [11,14,38]. Consistent with our results, Smith et al. observed a substantial increase in the collagen isoforms, I, III, IV, and VI, in the ST muscle of CP children compared to typically developing children [38], while Pingel et al. found a statistically significant positive correlation between passive muscle stiffness and gene transcription of *COL4A6* (*collagen type IV alpha-6 isoform*) in the MG of CP patients [13]. Interestingly, a study on transgenic SOD1G93A mice expressing an amyotrophic lateral sclerosis (ALS) phenotype demonstrated augmented deposits of collagen I and III in the gastrocnemius muscle upon manifestation of the pathological ALS phenotype [39]. Studies involving individuals with CP and mice exhibiting an ALS phenotype thus suggest that the different collagen isoforms in the ECM, including collagen type IV, may play a role in muscle dysfunction following damage to the upper motor neurons.

Another aspect worth considering is the extent to which the predominant muscle fiber type may affect ECM remodeling after TBI, or whether a brain injury could potentially modify the composition of muscle fibers. A study using a controlled cortical impact method on mice suggested a connection between atrophy of the contralateral SOL muscle fibers and the muscle’s primary fiber type [40]. Given that BF, EDL, LG, MG, ST, and VL muscles in rats are predominantly composed of type II fibers [41], and that these were the primary muscles showing an increase in laminin and/or collagen IV in this study, this prompts speculation regarding the potential influence of the dominant muscle fiber type on ECM modification after TBI. However, in our study, PL did not exhibit an increase in ECM protein expression, while SOL, which is primarily composed of type I fibers in rats [41], showed an increased ECM protein expression. This means that such a speculation cannot stand alone.

In CP children, it appears that the changes in the ECM, rather than the changes in muscle fibers, contribute to the increased stiffness and contracture in spastic muscles [11]. Thus, this should also be considered in relation to our study: Is the increased expression of collagen IV and laminin in the ECM of hindlimb muscles the cause of the development of HL-PA following TBI? As the muscle relaxant pancuronium completely eliminated HL-PA in TBI rats [4], this may not be the case. It is more likely that the reduced muscle activity after TBI induces the remodeling of the muscle ECM. This suggestion is supported by the finding that immobilization following TBI could contribute to an altered ECM composition. It has previously been shown that there is a correlation between an increased total collagen content in SOL and a declined range of motion of the dorsiflexion after three weeks of immobilization in rats [42]. It is also possible that, since the increased expression of laminin and collagen IV was found both in flexor and extensor hindlimb muscles following a unilateral TBI, the impact of ECM on the formation of HL-PA might not manifest. It is plausible to speculate that changes in the ECM within the hindlimb muscles may underpin various kinematic deficits observed following TBI, such as alterations in the gate pattern [3].

Our study possesses some limitations. Firstly, the numbers of the animals in each group were small. Increasing the sample size in both groups would have facilitated more robust statistical analyses. Secondly, the study relied on semi-quantitative analyses based on immunohistochemistry and light microscopy. Conducting quantitative analyses, such as ELISA, Western blot, or liquid chromatography with tandem mass spectrometry, would enhance the study’s quality and enable a more precise detection of changes in protein expressions. Comparative measurement of the mRNA levels for the investigated ECM proteins would also have been beneficial. Thirdly, to explore potential differences in the expression of ECM in hindlimb muscles, it would have been useful to include TBI on the right side for comparison. Lastly, it is also important to recognize that our TBI model targets a specific brain area, which may not align with the clinical heterogeneity observed in TBI patients differing from the wide range of injury sites seen in clinical TBI cases, where injuries seldom are confined to a singular region [43,44]. Despite these aspects, our rat TBI model remains a valuable tool for exploring the underlying pathological mechanisms of motor deficits focusing on a distinct and localized area of the cerebral cortex.

In conclusion, our study has demonstrated that a focal TBI can lead to the development of contralateral hindlimb flexion along with fragmentation and disruption of laminin and collagen IV in the hindlimb muscles from both sides and an elevated expression of laminin and collagen IV in different contralateral hindlimb muscles. It still needs to be further investigated whether flexion of the contralateral hindlimb is a cause or a consequence of ECM remodeling following TBI. Our findings also suggest a similarity in the processes related to ECM changes occurring in both CP and TBI as suggested by other researchers [45]. This could be attributed to the immobility of the affected muscles or other yet unidentified mechanisms. These alterations may ultimately contribute to muscle contracture in these neurological conditions.

## Figures and Tables

**Table 1:** Overview of the collected muscles, their functions as flexors and/or extensors, and the joints to which they connect.

Muscle	Function	Acting Joint
Muscle from the leg:		
Extensor digitorum longus (EDL)	Dorsal flexion	Ankle
Peroneus longus (PL)	Plantar flexion and eversion	Ankle
Lateral head of gastrocnemius (LG)	Flexion Plantar flexion	Knee Ankle
Medial head of gastrocnemius (MG)	Flexion Plantar flexion	Knee Ankle
Soleus (SOL)	Plantar flexion	Ankle
Muscle from the thigh:		
Biceps femoris (BF)	Flexion Extension	Knee Hip
Semitendinosus (ST)	Flexion Extension	Knee Hip
Vastus lateralis (VL)	Extension	Knee

## Data Availability

The data presented in this study are available on request from the corresponding author.

## Supplementary Materials

The following supporting information can be downloaded at: https://www.mdpi.com/article/10.3390/life14050543/s1, Figure S1: Correlation analyses between HL-PA and the expression changes of laminin or collagen IV in TBI rats show no significant correlations between the two parameters in any of the investigated hindlimb muscles (*p* > 0.05).
