# Supplementary material for: Focal Traumatic Brain Injury Impairs the Integrity of the Basement Membrane of Hindlimb Muscle Fibers Revealed by Extracellular Matrix Immunoreactivity
Source: Life (Basel). 2024 Apr 24;14(5):543. doi: 10.3390/life14050543 (PMC11121831; doi:10.3390/life14050543)
Supplement: Supplementary file 1 [file life-14-00543-s001.zip › life-2892806-supplementary.pdf]

Supplementary Figure S1

Kristensen et al., Life, 2024

Laminin

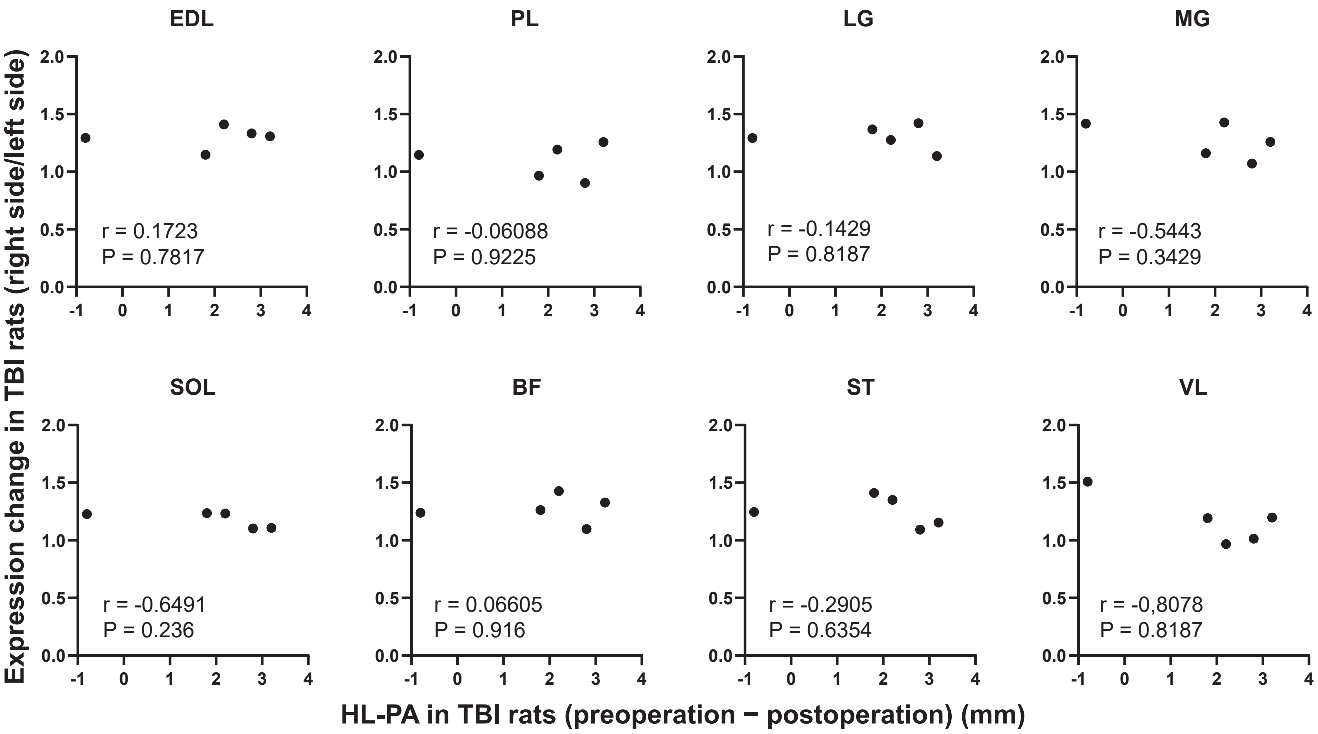

Collagen IV

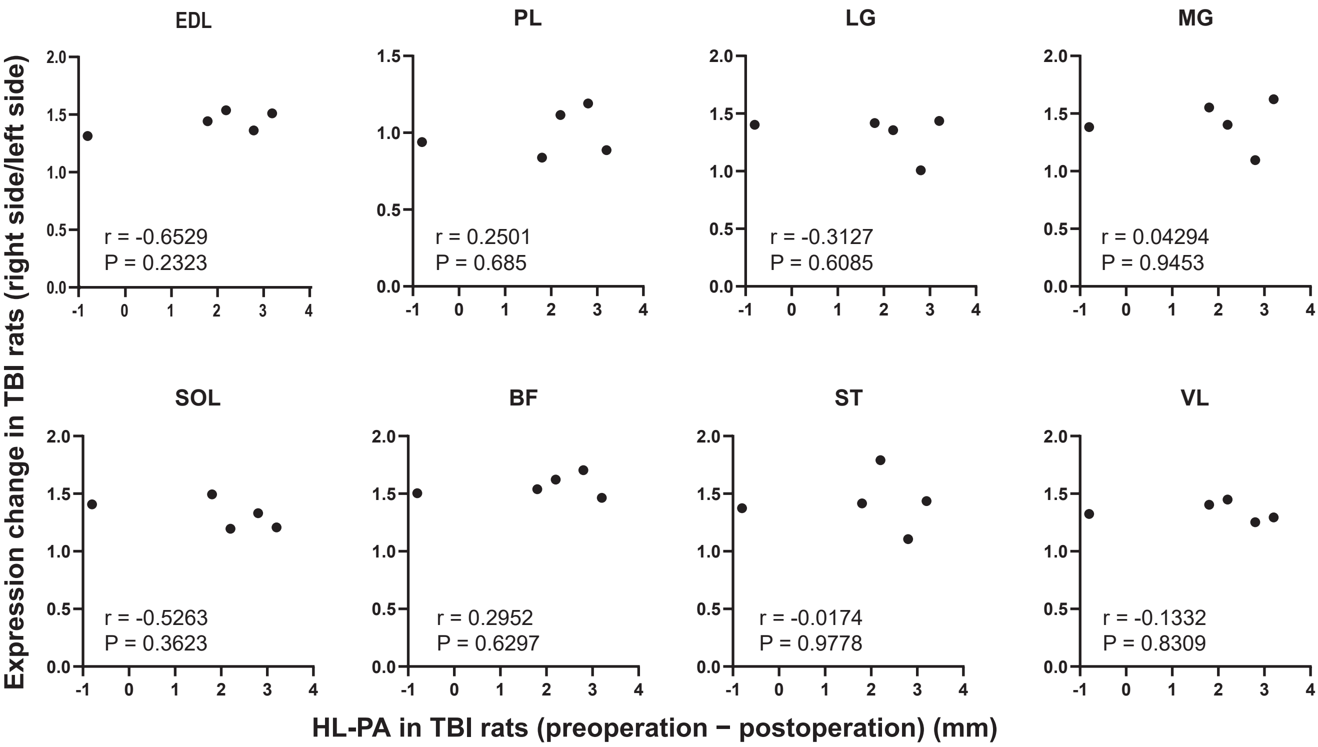

Supplementary Figure S1. Correlation analyses between HL-PA and the expression changes of laminin or collagen IV in TBI rats show no significant correlations between the two parameters in any of the investigated hindlimb muscles (P > 0.05).
